# Photoplethysmography in Diverse Skin Tones: Evaluating Bias in Smartwatch Health Monitoring

**DOI:** 10.7759/cureus.94074

**Published:** 2025-10-07

**Authors:** Salwa Asif, Abdallah AlSaafeen, Soumiya Nadar, Shristhi Nambiar, Jihad Dannawi, Naga Harika Korrapati, Harsahaj S Wilkhoo

**Affiliations:** 1 General Practice, Faculty of Medicine, Tbilisi State Medical University, Tbilisi, GEO; 2 Medical School, Faculty of Medicine, Tbilisi State Medical University, Tbilisi, GEO; 3 General Practice, Makassed General Hospital, Beirut, LBN; 4 Epidemiology and Public Health, University of New Haven, New Haven, USA

**Keywords:** heart rate monitoring, photoplethysmography, skin tone bias, smartwatches, wearable health technology

## Abstract

Heart disease remains one of the most pressing global health burdens, and the growth of wearable technology has introduced new possibilities for prevention and monitoring. Smartwatches commonly use photoplethysmography (PPG) to estimate heart rate by detecting changes in light reflected from the skin. However, melanin’s absorption of green light raises concerns about measurement accuracy in individuals with darker skin tones. This narrative review, conducted in accordance with the PRISMA extension for narrative reviews, examined studies published between May 2017 and May 2025. Searches were performed in PubMed, Google Scholar, and the American College of Cardiology (ACC) databases, yielding 50 records, of which 23 met the inclusion criteria. Only English-language studies were included, and duplicates were removed through automated and manual screening. Findings demonstrated significant variability across devices. One validation study of 60 participants reported that a major smartwatch brand showed mean heart rate differences of less than 5 bpm across skin tones (95% CI: -3.2 to +4.1). By contrast, other brands underestimated heart rate by 10-15 bpm at rest and by more than 20% during vigorous activity in darker-skinned users (n = 75, p < 0.01). Devices using alternate operating systems displayed inconsistent calibration, with error rates reaching up to 12%. Large-scale studies involving over 400,000 participants reported greater than 95% sensitivity for atrial fibrillation detection, though stratification by skin tone was not conducted. Skin-tone-related bias in smartwatch PPG sensors poses risks that extend beyond fitness tracking to arrhythmia detection, hypertension monitoring, and telehealth reliability. To improve equity, standardized validation protocols stratified by Fitzpatrick skin type and activity level are essential, alongside regulatory mandates for inclusive testing. Future research should include longitudinal studies across diverse cohorts to ensure wearable health technologies become reliable tools for all populations.

## Introduction and background

**Figure 1:**
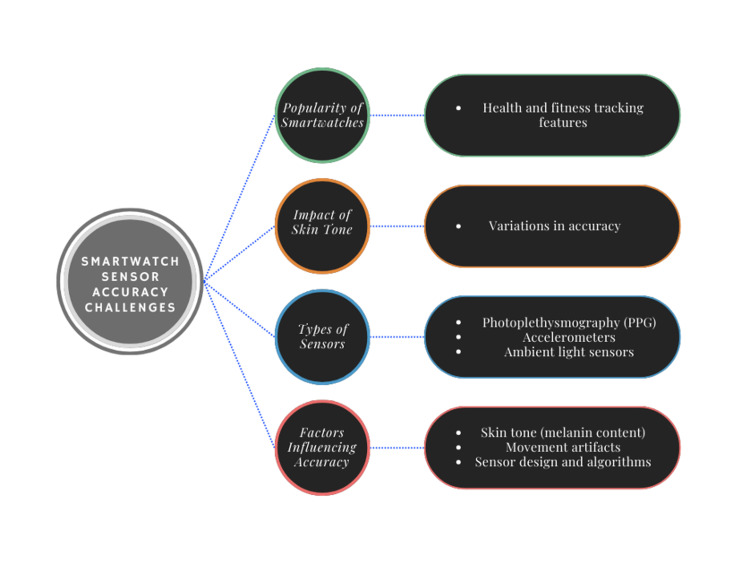
Flowchart of smartwatch accuracy challenges, showing how skin-tone–related bias influences PPG signal quality, sensor hardware, and algorithmic processing. Illustrated by Asif S.

Skin tone can affect the accuracy of heart rate sensors in smartwatches, which are often used to check fitness and health, as shown in Figure 1. Photoplethysmography (PPG) technology typically relies on green light wavelengths, which are particularly affected by melanin’s higher absorption [1]. This makes darker skin tones more susceptible to signal distortion and data loss compared to lighter skin [2]. As darker skin absorbs and scatters lighter light, melanin levels can change PPG sensors, which measure blood flow through the skin, producing inaccurate results [3]. This reduces the reliability of the data by either overestimating or underestimating heart rate [4]. While some manufacturers have attempted to adjust algorithms to improve accuracy, evidence suggests that these modifications have not consistently eliminated bias, and error rates are still reported across different skin tones in recent studies. Studies continue to reveal variations in heart rate measurement for individuals with darker skin, despite manufacturers' efforts to address this issue by creating sensors and algorithms. Therefore, proving the reliability of wristwatch data is essential for making well-informed health decisions [5,6]. Clinically, inaccurate smartwatch readings can have serious consequences. Reduced accuracy in darker skin tones has been linked to missed detection of atrial fibrillation when irregular pulse notifications fail to capture abnormal rhythms. Errors during exercise monitoring may result in inappropriate training intensities or misleading recovery assessments [7,8,9]. These findings underscore the need to address skin tone bias in wearable health technologies to prevent misdiagnosis and ensure equitable care.

With their combination of ease of use, technological prowess, and visual appeal, smartwatches have emerged as the most popular type of wearable technology. Real-time monitoring and improved patient management have been made possible by smart wearable technology, revolutionizing the healthcare industry. Fitness trackers like Fitbit (Fitbit, Inc., San Francisco, CA) and Samsung Gear Fit (Samsung Electronics, Suwon, South Korea), for instance, can measure heart rate, sleep patterns, and physical activity to give users useful information about their health [9,10]. Additionally, by enabling users to send emergency notifications, smart accessories such as necklaces and bracelets with Cufflinks technology improve personal safety [11,12].

Interestingly, 95% of wearable device usage is on smartwatches, showing their significance in this space [13-16]. More recent reports indicate that smartwatch usage has continued to rise steadily through 2023-2024, with global shipments surpassing 150 million units annually and adoption rates exceeding 30% of adults in the United States and Europe. The adoption statistics presented in Table 1 are derived from global shipment data and should be interpreted as representing worldwide populations. However, regional variations may exist, and socioeconomic disparities in access to these devices may further interact with skin-tone-related bias, raising additional equity concerns [17-23]. Moreover, socioeconomic disparities in access to these devices may further interact with skin-tone-related bias, raising additional equity concerns [17-23]. Consequently, there was a notable surge in worldwide Apple smartwatch (Apple Inc., Cupertino, CA) shipments from 2017 to 2019, as indicated in Table 1, and a sharp rise in smartwatch usage from 2013 to 2018.

**Table 1:** Epidemiology and prevalence of smart watch usage Adapted from references [20-23]

Metric	Statistics/Findings
Global adoption rate	Smartwatches account for 95% of wearable device worldwide
Growth in U.S. usage	Adoption in the US rose to 14% between 2013 and 2018
Healthcare monitoring	Widely used to track heart rates, sleep patterns and seizures
Age demographics	Most users are between 18-34, drawn by function and style
Gender adoption	Slightly higher usage among males, though smartwatch designs for female users are gaining popularity

## Review

Methods

Search Approach

**Figure 2:**
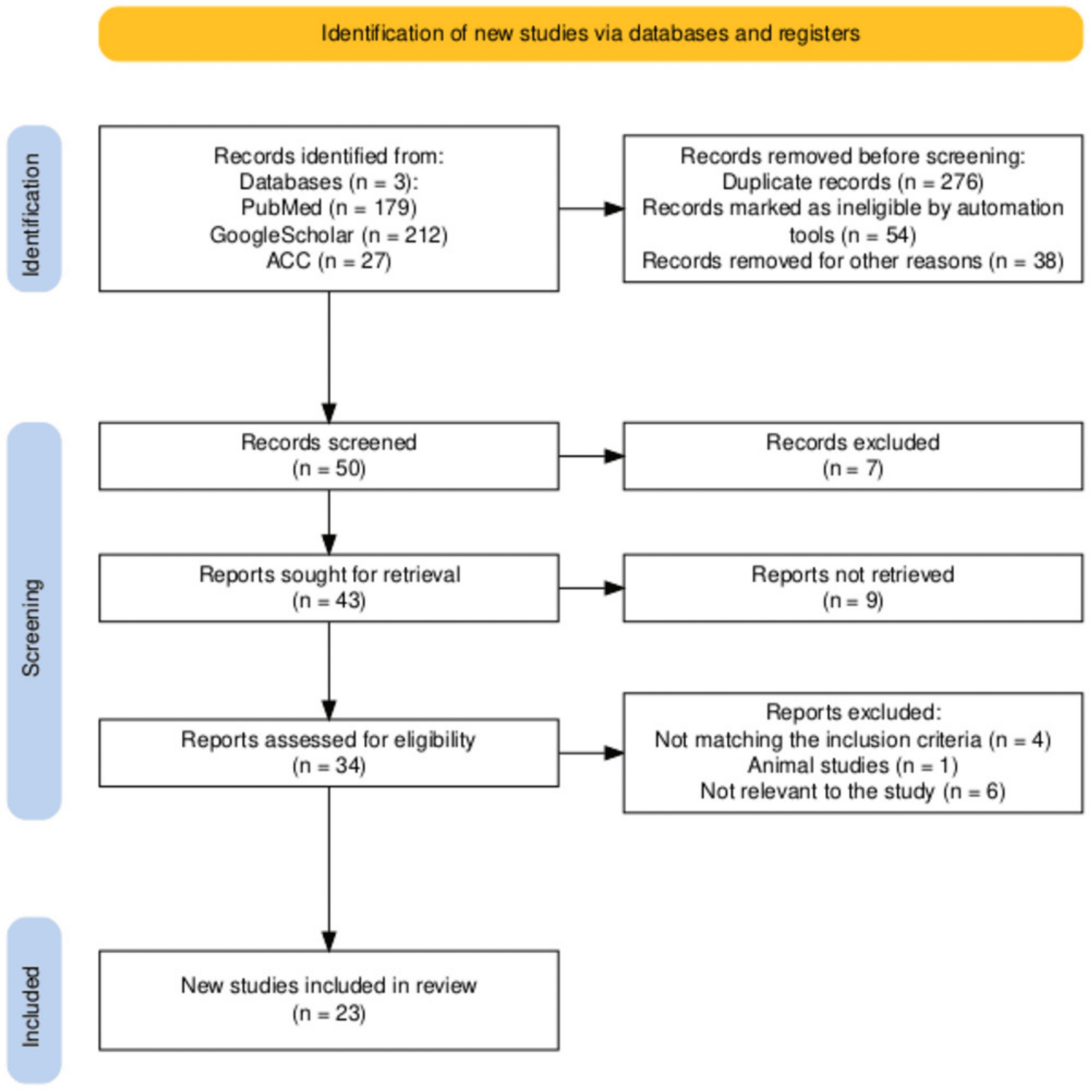
PRISMA Flowchart ACC: American College of Cardiology

This narrative review was conducted in accordance with PRISMA guidelines for narrative syntheses and literature search with appropriate strategies, inclusion, and exclusion criteria (Figure 2). Relevant literature was identified through databases such as PubMed, Google Scholar, and the American College of Cardiology (ACC), covering the period from May 2017 to May 2025. Key search terms included “smartwatches,” “health monitoring,” “skin tone,” “racial bias,” and “PPG.” Only English-language publications were considered. Non-English articles, gray literature, and conference abstracts were excluded.

Eligibility Criteria

Fifty records were initially identified. After screening, 23 studies were included for detailed review. Duplicate and overlapping studies were removed using reference management software, with manual verification to ensure accuracy. Furthermore, inclusion and exclusion criteria are summarized in Table 2.

**Table 2:** Inclusion and Exclusion Criteria PPG, photoplethysmography

Inclusion Criteria	Exclusion Criteria
Studies evaluating smartwatch-derived health or heart rate data across different skin tones	Duplicate or overlapping datasets
Use of PPG or optical heart rate monitoring	Studies without stratification by skin tone/pigmentation
Clear reporting of accuracy outcomes, error rates, or bias-related findings	Case reports or studies lacking methodological detail
Peer-reviewed, English-language publications	Non-peer-reviewed sources, gray literature, or conference proceedings

Study Selection and Data Synthesis

A total of 50 publications were identified across PubMed, Google Scholar, and ACC. After screening, 43 reports were sought for retrieval, with 34 assessed for eligibility. Eleven studies were excluded (four not meeting inclusion criteria, one animal study, six not relevant). Finally, 23 studies were included in the narrative synthesis.

Data were narratively synthesized and thematically grouped into sensor accuracy, algorithmic calibration, clinical implications, and equity considerations. The selection process based on PRISMA guidelines is shown in Figure 2.

Results

Studies consistently demonstrated that smartwatch accuracy is influenced by skin tone, though the magnitude of this effect varied widely across devices and contexts (Table 3). Separate from these skin-tone-focused findings, some included studies provided broader insights into wearable technology more generally. For example, Henriksen et al. (2018) summarized validation trends across 423 devices [8], while Dias et al. (2018) [4] and Castaneda et al. (2018) [20] discussed wearable sensor architecture and system design. Although not stratified by skin tone, these studies add technological context but were interpreted cautiously in relation to pigmentation bias. In PPG-based measurements, melanin absorption of green light was identified as a primary factor interfering with optical signal detection [9]. Ray et al. (2021) observed that some WearOS smartwatches underestimated heart rate by 10-15 bpm in darker-skinned users during moderate to vigorous exercise, compared with near-baseline error in lighter-skinned participants [1]. In contrast, Apple Watch devices were more consistent, with <5 bpm variation across skin tones, findings supported by Sañudo et al. (2019) and Shcherbina et al. (2017) [7,10].

**Table 3:** Overview of wearable health device studies (2013–2025): study design and main findings Abbreviations: API, application programming interface; BCT, behavior change technique; FBG, fiber Bragg grating; FFT, fast Fourier transform; FST, Fitzpatrick skin type; HR, heart rate; HRV, heart rate variability; PPG: photoplethysmography

Author	Study	Findings
Ray I et al., 2021	Observational study	Some WearOS smartwatches reduce heart rate data for darker-skinned users despite similar accuracy across skin tones. Inconsistent API use across brands affects data reliability [1].
Colvonen PJ et al., 2020	Review article	Green light-based wearables underperform on darker skin due to light absorption. The study urges inclusive validation to avoid worsening health disparities [2].
Huhn S et al., 2022	Review article	Consumer wearables are increasingly used for health monitoring, with common metrics including heart rate and sleep. The study emphasizes the potential of big data from these devices [3].
Dias D et al., 2018	Review article	Reviews wearable health tech evolution, focusing on vital sign monitoring and system architecture. Highlights use of smart garments and commercial devices [4].
Bent B et al., 2020	Observational study	PPG-based monitors show similar accuracy across skin tones at rest but increased error during activity. Highlights need for context-aware use [5].
Sañudo B et al., 2019	Comparative study	Apple Watch shows high HR accuracy during exercise with minimal variation across skin tones. Slight underestimation considered clinically negligible [7].
Koerber D et al., 2023	Systematic review	The study finds mixed evidence on whether skin tone affects heart rate accuracy from wearables, highlighting the need for more robust research with larger, more diverse samples [9].
Shcherbina A et al., 2017	Observational study	This study assessed heart rate and energy expenditure accuracy across several wearables, finding minimal HR error but significant discrepancies in energy expenditure, particularly during walking and cycling [10].
Reddy RK et al., 2018	Observational study	The study found that Fitbit Charge 2 and Garmin vívosmart HR+ showed reasonable heart rate accuracy, but both struggled during high-intensity activities like cycling [11].
Ware OR et al., 2020	Review article	A survey of dermatologists found the Fitzpatrick Skin Type (FST) scale is widely used to assess skin color, though its original purpose should be emphasized in medical education [12].
Xiao K et al., 2017	Systematic review	The study explores ethnic skin color differences, revealing significant variations in redness and yellowness across different ethnic groups, influencing medical and cosmetic applications [13].
Zhang Y et al., 2020	Systematic review	The study finds wrist-worn heart rate devices to be accurate for typical activities but less reliable for activities like resistance training and cycling [14].
Perez MV et al., 2019	Observational study	The study proved that smartwatch irregular pulse notifications can accurately show atrial fibrillation in a large-scale, site-less monitoring approach [15].
Haxha S et al., 2024	Observational study	The study showed that fingertip location and skin tone had no significant clinical impact on pulse oximeter readings across commercial and custom devices [16].
Li K et al., 2023	Observational study	The study explores the potential of heart rate variability (HRV) tracking with wearable devices, aiming to enhance personal health monitoring and disease diagnosis [17].
Shi C et al., 2023	Observational study	Introduces a high-precision cardiovascular pulse sensor based on fiber Bragg grating (FBG), showing low error and high sensitivity for wearable devices [18].
Castaneda D et al., 2018	Review article	Highlights the use of photoplethysmography (PPG) for heart rate monitoring and its potential for detecting cardiovascular diseases, despite ongoing challenges in technology [20].
Duncan M et al., 2017	Observational studies	Finds variability in behavior change techniques (BCTs) among devices, with a stronger focus on physical activity compared to sleep or sedentary behavior [22].
Nelson BW et al., 2020	Observational studies	Discusses the use of consumer wearables for checking cardiovascular psychophysiological processes, showing issues with data standardization and participant demographics that compromise accuracy [23].
Ibtehaz N et al., 2022	Experimental study	Introduces PPG2ABP, a noninvasive method for continuous arterial blood pressure monitoring using deep learning, achieving high accuracy, and meeting clinical standards [24].
Ferreira ND et al., 2021	Review article	Reviews heart rate as a key cardiac health indicator, emphasizing the challenges of motion artifacts in wrist-worn devices and the need for continuous monitoring in ambulatory settings [25].

Bent et al. (2020) reported that PPG devices showed error rates exceeding 20% during cycling and resistance exercise in individuals with higher pigmentation, while maintaining <10% error at rest, underscoring the importance of activity context [5]. Comparisons across brands also revealed that Garmin vívosmart HR+ (Garmin Ltd., Olathe, KS) and Fitbit Charge 2 were shown to achieve reasonable heart rate accuracy during steady-state activity but produced large deviations during cycling, particularly in darker-skinned users. By contrast, Apple devices tended to perform better during exercise but still slightly underestimated peak values, a discrepancy deemed clinically negligible [9].

Koerber et al. (2023), in a systematic review, emphasized this heterogeneity, concluding that no single brand was consistently superior across all skin tones and activities [9]. When stratified by outcomes, exercise-based heart rate monitoring was most affected by skin-tone bias, while resting heart rate accuracy was generally preserved [12-14]. Importantly, arrhythmia detection algorithms such as the irregular pulse notification in the Apple Heart Study (Perez et al., 2019) remained robust, with sensitivity for atrial fibrillation unaffected by skin tone across a large-scale population [15].

On the other hand, heart rate variability (HRV) applications showed promise but remain underexplored in terms of pigmentation differences [16]. Li et al. (2023) demonstrated reliable HRV tracking overall but did not stratify outcomes by skin tone [17]. Additionally, Dias et al. (2018) and Castaneda et al. (2018) highlighted the potential of red and infrared light wavelengths, which are less absorbed by melanin, to reduce error rates and enhance inclusivity [16,17]. Experimental prototypes, such as fiber Bragg grating (FBG)-based pulse sensors (Shi et al., 2023), achieved low error margins (<2%) across diverse skin types, suggesting future design directions [18].

Discussion

The precision of smartwatches depends heavily on skin tone since accurate wearable health measurements require reliable data. Health tracking through wristwatches will require further investigation because their growing popularity necessitates a complete understanding of this limitation. Photoplethysmography (PPG) detects heart rate by measuring light absorption and reflection occurring within the skin's microvascular bed during blood pulses according to [20]. PPG sensors generate heart rate measurements by applying LEDs to measure skin-based light reflection through photodetectors that evaluate changes in intensity [21]. Skin pigmentation affects light absorption and reflection rates such that darker skin tones reduce sensor signal detection capabilities while increasing misread errors. As melanin absorbs more light, darker skin reduces PPG signal strength, leading to weaker detection. However, findings across the literature are conflicting: while Apple Watch studies reported minimal error (<5 bpm) regardless of skin tone, other brands such as WearOS showed underestimations of 10-15 bpm, and certain devices (e.g., Fitbit, Garmin) recorded error rates exceeding 20% during cycling in darker-skinned participants [22-25]. This demonstrates that discrepancies are not uniform and may depend on device, algorithm, and activity context.

Studies have established that smartwatch heart rate calculations function less accurately on individuals with dark skin tones, suggesting improvements must be made to technology in order to close these performance gaps. Beyond fitness tracking, the clinical implications are broader: smartwatch-derived atrial fibrillation notifications have been validated in large-scale cohorts [25], yet blood pressure monitoring, hypertension surveillance, and telehealth systems relying on wearable data could be jeopardized if measurement bias persists. Clinically meaningful error is typically considered >10 bpm in cardiology monitoring, meaning even modest inaccuracies may alter clinical decision-making. Mitigation strategies, including red and infrared light wavelengths or multimodal sensors that combine optical with accelerometer data, have shown potential in reducing pigmentation-related error. Despite these developments, regulatory authorities such as the FDA and CE currently lack explicit requirements for validating wearable performance across diverse skin tones, raising concerns for health equity. Long-term impacts of repeated underestimation in darker-skinned populations - such as chronic underdiagnosis of cardiovascular risk - remain largely unexplored, underscoring the need for standardized validation protocols and inclusion of diverse demographic groups in future studies [26]. Misleading heart rate readings lead to several negative consequences, such as substandard recovery assessments and improper exercise intensity control, and cardiovascular condition identification, resulting in deteriorated fitness results and healthcare outcomes [27]. Theoretical implications include flawed training load assessment, stress evaluation, and recovery modeling, whereas real-world outcomes documented in clinical contexts involve misclassification of exercise intensity and delayed arrhythmia detection. Distinguishing between these dimensions helps clarify both potential risks and observed clinical consequences. For example, Bent et al. (2020) quantified error >20% during cycling in darker-skinned individuals compared with <10% at rest, showing how context alters reliability [5].

Heart rate measurement accuracy is influenced by skin tone due to variations in light absorption, reflection, and penetration, which affect PPG signal quality [18]. Darker skin, with higher melanin levels, absorbs more light, reducing signal strength and limiting light penetration to blood vessels [19]. This results in weaker signals and increased scattering, which degrade measurement accuracy [19,20]. In contrast, lighter skin reflects more light, allowing better signal detection. Many sensors lack algorithms to effectively separate weak physiological signals from background noise in darker skin tones, further lowering accuracy [20]. Comparative evaluations indicate that Apple and Fitbit’s proprietary algorithms outperform smaller-brand or open-source counterparts, largely because of superior noise filtering and adaptive calibration [9,11]. However, limited transparency in algorithm design prevents external validation, which remains a major obstacle. Studies show that smartwatches often emit insufficient light for accurate readings on darker-skinned users, leading to inconsistent results and potential misdiagnosis. These discrepancies undermine trust in wearable health devices, especially when inaccurate readings affect fitness tracking or miss critical signs like arrhythmias [21,22].

The accuracy of heart rate measurements affects people with darker skin tones because of algorithm developmental biases that stem from selecting features and adjusting training data composition and model calibrations [23-26]. Technological limitations are primarily sensor-related - such as LED wavelength, light intensity, and placement - while algorithmic limitations stem from biased datasets, poor calibration, and underrepresentation of darker skin in training cohorts. Separating these dimensions shows that hardware solutions (infrared/red-light sensors, multimodal approaches) and software solutions (AI/ML trained on diverse datasets) must progress in parallel to close the performance gap [27,28]. Integrating larger, more diverse training datasets with adaptive AI systems capable of real-time adjustment may reduce discriminatory biases and enhance equity of performance across populations. A majority of skin tone-related algorithmic models work best when applied to diagnostic data that includes patients with lighter complexions, while generating inferior outcomes for patients with darker complexions. The problem arises primarily from model biases as well as from issues with feature selection and calibration because better settings for lighter skin types create unequal outcomes for people with darker skin [29]. The combination of extensive training data with adaptive analytical systems capable of recognizing individual characteristics and providing real-time responses can minimize errors and discriminatory biases [29].

It is vital to establish the skin tone limits that influence smartwatch accuracy performance. The information will help both consumers who need to make technological choices and manufacturers who need to resolve wearable technology discrepancies [30]. Misleading heart rate information results in health management errors regarding recovery phases and fitness areas to produce substandard exercise programs and performance objectives [31]. The use of secondary measurement methods, including heart rate variability (HRV) derived from heart rate values, produces assessments that can be inaccurate for stress and recovery evaluation [32]. People with darker skin tones might experience poorer heart rate measurement accuracy because of their skin tones potentially skewing data used to develop research findings and clinical recommendations [33]. Healthcare professionals need to grasp these restrictions when they use wearable data to achieve better diagnostic and therapeutic results [34].

## Conclusions

This review highlights how melanin’s influence on light absorption and reflection contributes to systematic inaccuracies in smartwatch PPG, disproportionately affecting individuals with darker skin tones. Although adaptive algorithms and machine learning models are beginning to be explored, their validation remains inconsistent, and multi-wavelength approaches, such as red and infrared light, face trade-offs related to cost, battery consumption, and device design. The clinical implications of these inaccuracies extend beyond fitness tracking to critical domains, including arrhythmia detection, hypertension monitoring, and telehealth assessments, where even modest errors can alter diagnostic and therapeutic decisions. To mitigate these risks, standardized validation protocols should incorporate stratification by Fitzpatrick skin type, evaluation across varying activity levels and real-world contexts, and transparent regulatory oversight from agencies such as the FDA and CE.

The long-term impacts of underestimation in darker-skinned populations remain largely unexplored, highlighting the need for standardized validation and inclusive device testing. Future research should include longitudinal studies that assess health outcomes across diverse cohorts while addressing the urgent ethical concerns surrounding algorithmic bias and unequal access. It is recommended that manufacturers aim to provide clear user education to help prevent misinterpretation of device outputs. Achieving equitable accuracy in wearable health technologies, therefore, requires sustained collaboration among researchers, developers, regulators, and clinicians to ensure that innovation serves as a tool for inclusion rather than a vector for reinforcing disparities.
